# YTHDC2 Promotes Malignant Phenotypes of Breast Cancer Cells

**DOI:** 10.1155/2022/9188920

**Published:** 2022-10-07

**Authors:** Atsushi Tanabe, Takatoshi Nakayama, Jo Kashiyanagi, Hitoshi Yamaga, Yoshihiko Hirohashi, Toshihiko Torigoe, Fukino Satomi, Hiroaki Shima, Hideki Maeda, Goro Kutomi, Ichiro Takemasa, Hiroeki Sahara

**Affiliations:** ^1^Laboratory of Biology, Azabu University School of Veterinary Medicine, 1-17-71 Fuchinobe, Chuo-ku, Sagamihara, Kanagawa 252-5201, Japan; ^2^Department of Pathology, Sapporo Medical University School of Medicine, South 1 West 17, Chuo-ku, Sapporo 060-8556, Japan; ^3^Department of Surgery, Sapporo Medical University School of Medicine, South 1 West 17, Chuo-ku, Sapporo 060-8556, Japan

## Abstract

YTH domain-containing 2 (YTHDC2) is known to be an important regulator for RNA metabolism. Here, we show that YTHDC2 is essential for breast cancer tumorigenesis and metastasis. We examined YTHDC2 expression levels by immunohistochemistry in human breast tumor tissues from 99 patients and found a significantly positive correlation between the YTHDC2 expression level and the tumor stage. We established YTHDC2-knocked-down cell lines using four breast cancer cell lines with different subtypes. Knockdown of YTHDC2 attenuated the sphere-forming and the metastatic ability of breast cancer cells. Although stemness and EMT markers, such as SOX2, c-MYC, and NANOG, were downregulated in several YTHDC2-knocked-down breast cancer cells, a common target gene of YTHDC2 in breast cancer cells was not identified. These findings suggest that while YTHDC2 is involved in malignant progression of breast cancers, the mechanism by which YTHDC2 regulates those phenotypes is different between subtypes of breast cancers.

## 1. Introduction

YTH domain-containing 2 (YTHDC2) is a member of the YTH family of proteins, which has been shown to regulate a variety of mRNA metabolisms, including splicing, export, degradation, and translation [1, 2]. YTH family proteins specifically recognize N6-methyladenosine- (m^6^A-) modified mRNAs [1, 2]. m^6^A is a major posttranscriptional RNA modification that is reversibly regulated by methyltransferases and demethylases [3]. In addition to the YTH domain, YTHDC2 has an RNA helicase domain not found in other family proteins. The RNA helicase domain is thought to play a central role in the translation of target mRNAs by YTHDC2 [4]. Moreover, YTHDC2 is involved in the degradation of target mRNAs by interacting with exonuclease XRN1 [5, 6].

It has been reported that YTHDC2 plays important roles in various physiological and pathological processes. For instance, many studies have shown that YTHDC2 is deeply involved in germ cell differentiation [7–9]. YTHDC2 has been demonstrated to regulate the degradation and translation of meiotic transcripts in the early stage of meiosis [5, 7, 8]. YTHDC2 has also been shown to be involved in the replication of hepatitis C virus (HCV) [10, 11]. Kim and Siddiqui have demonstrated that YTHDC2 facilitates the translation of HCV RNA by binding to its internal ribosomal entry site [11]. Moreover, Zhou et al. have found that YTHDC2 suppresses liver steatosis by regulating the mRNA stability of lipogenic genes [12].

In tumorigenesis, YTHDC2 is also required for the translation of proteins that induce malignant phenotypes. We previously reported that YTHDC2 promotes colorectal cancer (CRC) progression by regulating the translation of hypoxia inducible factor 1*α* (HIF-1*α*) [13]. He et al. demonstrated that YTHDC2 is upregulated in radiation-resistant nasopharyngeal carcinoma and promotes radioresistance by activating the IGF1R/AKT/S6 signaling axis [14]. In addition, the upregulation of YTHDC2 expression has been observed in hepatocellular carcinoma (HCC), glioblastoma, and prostate cancer [15–18]. In contrast, the downregulation of YTHDC2 expression has been observed in head and neck squamous cell carcinomas and lung cancers [19, 20]. However, the biological functions of YTHDC2 remain unclear in many other types of cancer.

In this study, we investigated the roles of YTHDC2 in breast cancers. We found that the YTHDC2 expression level was positively correlated with the tumor stage. Knockdown of YTHDC2 suppressed the sphere-forming and metastatic activity of breast cancer cells. Moreover, several stemness transcription factors were downregulated in YTHDC2-knocked-down cells. Therefore, our findings indicate that YTHDC2 is involved in the malignant progression of breast cancers.

## 2. Materials and Methods

### 2.1. Immunohistochemistry (IHC) of Tumor Tissues from Breast Cancer Patients

**Figure 1:**
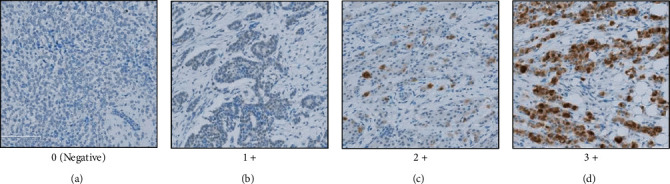
Representative immunostaining tissues with anti-YTHDC2 monoclonal antibody. Formalin-fixed paraffin-embedded tissues derived from breast cancer patients were stained with anti-YTHDC2 monoclonal antibody. Representative cases are shown in (a)–(d). (a)–(d) are determined YTHDC2-negative, 1+, 2+, and 3+ staining intensity, respectively.

Breast cancer tissue specimens (*n* = 99) that were resected from patients at Sapporo Medical University Hospital were used for the immunohistochemical examination. All tissues were obtained with written informed consent according to the guidelines of the Declaration of Helsinki and with approval of the Institutional Review Board of Sapporo Medical University Hospital (no. 282-134). Formalin-fixed paraffin-embedded tissue specimens were stained with anti-human YTHDC2 monoclonal antibody at a dilution of 1 : 100 and then detected using Bond Polymer Refine Detection (Leica Biosystems, Tokyo, Japan). The anti-YTHDC2 monoclonal antibody used for IHC was generated in our previous study [13]. Tumor tissues were evaluated as YTHDC2-positive with cytosol staining. Staining intensity of YTHDC2-positive tumor cells were evaluated for each case. Staining intensity scores were defined as 0 = no staining, 1+ = weak staining, 2+ = moderate staining, and 3+ = strong staining (Figure 1). All slides were reviewed and scored independently by three pathologists (pathology-trained doctor (HM) and certified pathologist (YH, TT)) in a blinded manner.

### 2.2. Cell Culture

The breast cancer cell lines MCF-7, MDA-MB-231, and MDA-MB-468 were cultured in Dulbecco's Modified Eagle Medium (Sigma-Aldrich, St. Louis, MO, USA), and the SK-BR-3 cell line was cultured in RPMI1640 medium (Sigma-Aldrich). Both media were supplemented with 10% fetal bovine serum, 200 unit/ml penicillin (Thermo Fisher Scientific, Waltham, MA, USA), 200 *μ*g/ml streptomycin (Thermo Fisher Scientific), and 2 mM L-glutamine (Thermo Fisher Scientific), and the cultures were maintained at 37°C in a humidified atmosphere with 5% CO_2_.

### 2.3. Short Hairpin RNA Transduction

Lentiviruses containing YTHDC2 short hairpin RNA (shRNA) or nontarget shRNA were purchased from Sigma-Aldrich. The YTHDC2 shRNA target sequence is 5′-GGAAGCTAAATCGAGCCTT-3′. The breast cancer cell lines were infected with the lentiviruses using 20 *μ*g/ml polybrene (Sigma-Aldrich). Each shRNA transductant was cultured in the presence of 1 *μ*g/ml puromycin and then cloned by limiting dilution, and stable YTHDC2-knocked-down (sh-Y2) and sh-control (sh-ct) cell lines were established.

### 2.4. Cell Proliferation Assay

For the cell proliferation assay, breast cancer cells were seeded in a 35 mm dish. These cells were trypsinized and counted using a hemocytometer after 2, 4, and 6 days. The culture medium was replaced every 2 days.

### 2.5. Sphere Formation Assay

MCF-7 was seeded at 1 × 10^4^ cells/well in an ultralow attachment 6-well plate (Corning Inc., Corning, NY, USA). SK-BR-3, MDA-MB-231, and MDA-MB-468 were seeded at 0.5 × 10^4^ cells/well in an ultralow attachment 6-well plate. These cells were cultured in tumor sphere medium (Takara Bio Inc., Shiga, Japan) for 5 days. Thereafter, we counted the sphere number of MCF-7 over 100 *μ*m diameter under low magnification. Since the shapes of spheres other than MCF-7 were very distorted, cell clusters with an average of minor axis and major axis of 100 *μ*m or more were counted.

### 2.6. Transwell Assay

We used transwell inserts with 8 *μ*m pores to assess the metastatic ability of the breast cancer cells. Cells were cultured in serum-free medium overnight and then harvested and resuspended in fresh serum-free medium. Next, 1 × 10^5^ cells in 0.2 ml of serum-free medium were placed on the upper chamber of the transwell inserts, and the lower chamber was filled with 0.8 ml of medium containing 10% fetal bovine serum. After 24 h of incubation, the cells in the upper chamber were gently removed with cotton swabs, and the cells that had migrated under the membrane of the transwell insert were stained with crystal violet. The migrated cells were then trypsinized and counted using a hemocytometer.

### 2.7. Metastasis Analysis Using a Nude Mouse Xenograft Model

Inbred, female BALB/c nu/nu mice (20-22 g, 6 weeks of age) were obtained from Japan SLC, Inc. (Shizuoka, Japan). All procedures were performed in compliance with the guidelines of the Animal Research Committee of Azabu University. MDA-MB-231 sh-Y2 or sh-ct cells were resuspended at 1 × 10^5^ cells per 50 *μ*l of PBS. Cells were injected into the exteriorized mammary glands after abdominal incision. At 6 weeks after cell injection, nude mice were sacrificed and lung metastasis examined by gross pathology.

### 2.8. Western Blotting

The primary antibodies used were as follows: anti-YTHDC2 (27779-1-AP) antibody was purchased from Proteintech Group Inc. (Rosemont, IL, USA); anti-E-cadherin (G-10), anti-vimentin (sc-6260), and cytokeratin 7 (sc-53263) antibodies were purchased from Santa Cruz Biotechnology, Inc. (Dallas, TX, USA); and anti-*β*-actin (A5441) antibodies were purchased from Sigma-Aldrich. First, 5 × 10^5^ cells were lysed in 100 *μ*l of RIPA lysis buffer (50 mM Tris-HCl pH 7.4, 150 mM NaCl, 1% Triton X-100, 1 mM ethylenediaminetetraacetic acid, 0.5% sodium deoxycholate, 0.1% sodium dodecyl sulphate, and protease inhibitor cocktail (Roche Applied Science)) for 30 min on ice. Then, 50 *μ*l of 3× sodium dodecyl sulphate sample buffer was added, and the samples were boiled at 100°C for 5 min. Next, the samples were subjected to 8% to 10% sodium dodecyl sulphate-polyacrylamide gel electrophoresis and were transferred onto a Hybond-ECL nitrocellulose membrane (Amersham Bioscience, Piscataway, NJ, USA). The transferred antigens on the membrane were detected by western blotting with the respective antibodies.

### 2.9. RNA Extraction and qRT-PCR Analysis

Total RNA was prepared using an RNeasy Mini Kit (QIAGEN Inc., Hilden, Germany) according to the manufacturer's instructions, and then, 500 ng of DNase I-treated total RNA was reverse-transcribed to cDNA with a Transcriptor First Strand cDNA Synthesis Kit (Roche Applied Science, Mannheim, Germany). Quantitative real-time PCR (qRT-PCR) reactions were performed with KAPA SYBR Fast Master Mix (KAPA Biosystems, Wilmington, MA, USA) using a Light Cycler 96 System (Roche Applied Science). qRT-PCRs were performed with the following cycling conditions: 95°C for 10 min; 45 cycles of denaturation at 95°C for 10 seconds, annealing at 60°C for 10 seconds, and extension at 72°C for 10 seconds, and then a dissociation step that consisted of 95°C for 0 seconds, 60°C for 15 seconds, and 95°C for 0 seconds to verify the presence of a single melting peak. The qRT-PCR primer sets used in this study are listed as follows: *YTHDC2* 5′-GGCATTCCCAATGACAGTAGTG-3′ and 5′-GCAATAAACTAGCTGCCTCTGG-3′; *OCT4* 5′-AGGTATTCAGCCAAACGACCA-3′ and 5′-GCACGAGGGTTTCTGCTTTG-3′; *SOX2* 5′-TACAGCATGATGCAGGACCA-3′ and 5′-CGAGCTGGTCATGGAGTTGTA-3′; *c-MYC* 5′-TCGGATTCTCTGCTCTCCTC-3′ and 5′-TGTTCCTCCTCAGAGTCGCT-3′; *KLF4* 5′-TCGGCCAATTTGGGGTTTTG-3′ and 5′-CAGGTGGCTGCCTCATTAATG-3′; *NANOG* 5′-CAGCTACAAACAGGTGAAGACC-3′ and 5′-TGCTATTCTTCGGCCAGTTG-3′; *TBP* 5′-TTGCTGCGGTAATCATGAGG-3′ and 5′-TTCTTCACTCTTGGCTCCTGTG-3′.

To correct for differences in both RNA quality and quantity between samples, each target gene was first normalized by dividing the copy number of the target by the copy number of *TBP*.

### 2.10. Statistical Analysis

Values are shown as the mean ± standard deviation. The Statcel 3 add-in (OMS Publishing, Saitama, Japan) for Microsoft Excel was used for the statistical analyses. The association between YTHDC2 expression level and clinicopathological parameters was determined using the Kruskal–Wallis test or Spearman's rank correlation coefficient. Data were analyzed by Student's *t*-test for comparisons between two groups and two-way analysis of variance followed by the Tukey-Kramer post hoc test for multiple comparisons. *P* values < 0.05 or <0.01 were considered to be statistically significant.

## 3. Results

### 3.1. The YTHDC2 Expression in Breast Tumor Tissues

We firstly investigated the correlation between breast cancer progression and YTHDC2 expression levels. We examined 99 patient specimens of breast tumor tissues by IHC. Representative immunostainings of breast tumor tissues showing 0, 1+, 2+, and 3+ staining intensity score for YTHDC2 are shown in Figures 1(a)–1(d). The YTHDC2 expression levels were positively correlated with the breast tumor stages (Table 1; Spearman *r* = 0.216; *P* = 0.032). On the other hand, no significant correlation was found between other clinicopathological factors, including molecular subtypes, and the YTHDC2 expression levels (Table 1). The YTHDC2 expression levels tended to correlate with the progression of breast cancer, suggesting that the YTHDC2 molecule may be extensively involved in the exacerbation of breast cancer.

### 3.2. Knockdown of YTHDC2 Expression Suppressed Cell Sphere-Forming Ability

**Figure 2:**
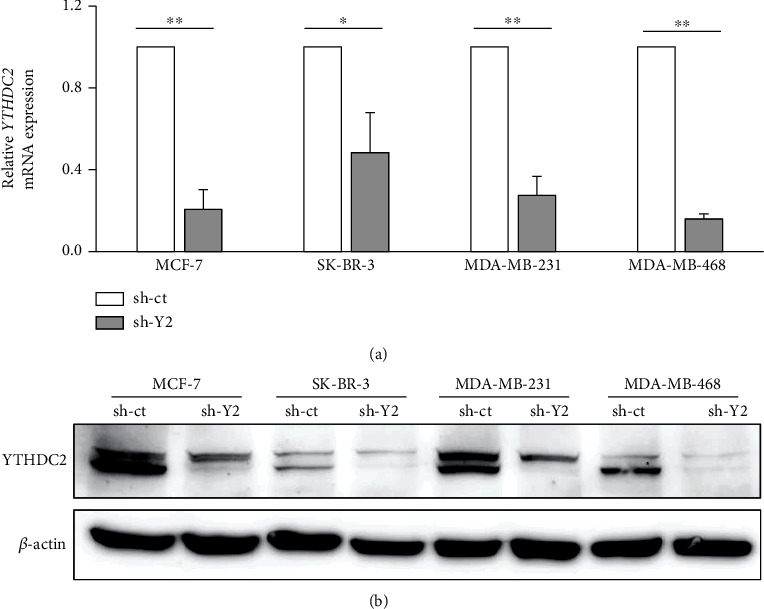
Stable knockdown of YTHDC2 in breast cancer cell lines. Breast cancer cell lines were transfected with control (sh-ct) or YTHDC2 (sh-Y2) shRNA. Knockdown efficiency of YTHDC2 was confirmed by (a) quantitative RT-PCR and (b) western blot analysis.

**Figure 3:**
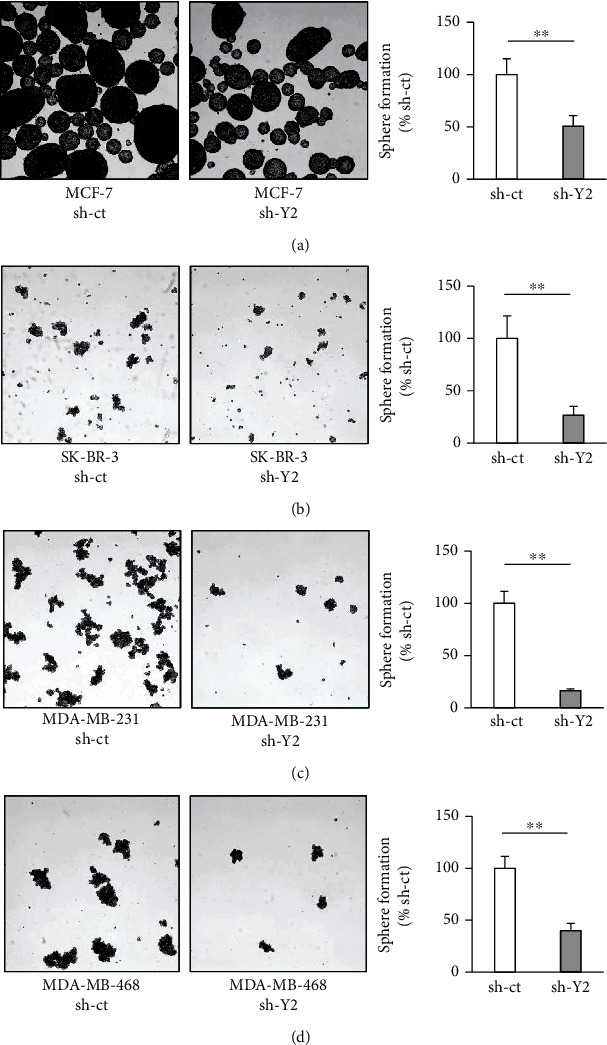
Effect of YTHDC2 knockdown on sphere-forming ability of breast cancer cells. Representative images of sphere-formed breast cancer cells: (a) MCF-7; (b) SK-BR-3; (c) MDA-MB-231; (d) MDA-MB-468. The data of bar graph are represented as the mean ± SD of the number of sphere-formed cells, and the results were analyzed using Student's *t*-test. ^∗^*P* < 0.05 and ^∗∗^*P* < 0.01 versus sh-ct.

To clarify the role of YTHDC2 in breast cancer, we knocked down the endogenous YTHDC2 expression by infecting MCF-7, SK-BR-3, MDA-MB-231, and MDA-MB-468 cells with lentivirus containing YTHDC2 shRNA (Figures 2(a) and 2(b)). The molecular subtypes of each cell line are classified into luminal A (MCF-7), HER2 positive (SK-BR-3), and triple negative (MDA-MB-231 and MDA-MB-468), respectively [21]. In some cases, MDA-MB-231 is classified as claudin-low type and MDA-MB-468 is classified as basal-like type [22]. We found that by knocking down YTHDC2, the cell proliferation rate of MCF-7 and MDA-MB-468 cells decreased (Figures S1A and S1D), while that of SK-BR-3 and MDA-MB-231 did not change (Figures S1B and S1C). We further evaluated tumorigenesis in the breast cancer cells *in vitro* by performing a sphere formation assay. We found that YTHDC2 knockdown significantly decreased the number of sphere-forming cells (Figures 3(a)–3(d)).

### 3.3. Knockdown of YTHDC2 Suppressed the Metastatic Ability of Breast Cancer Cells

**Figure 4:**
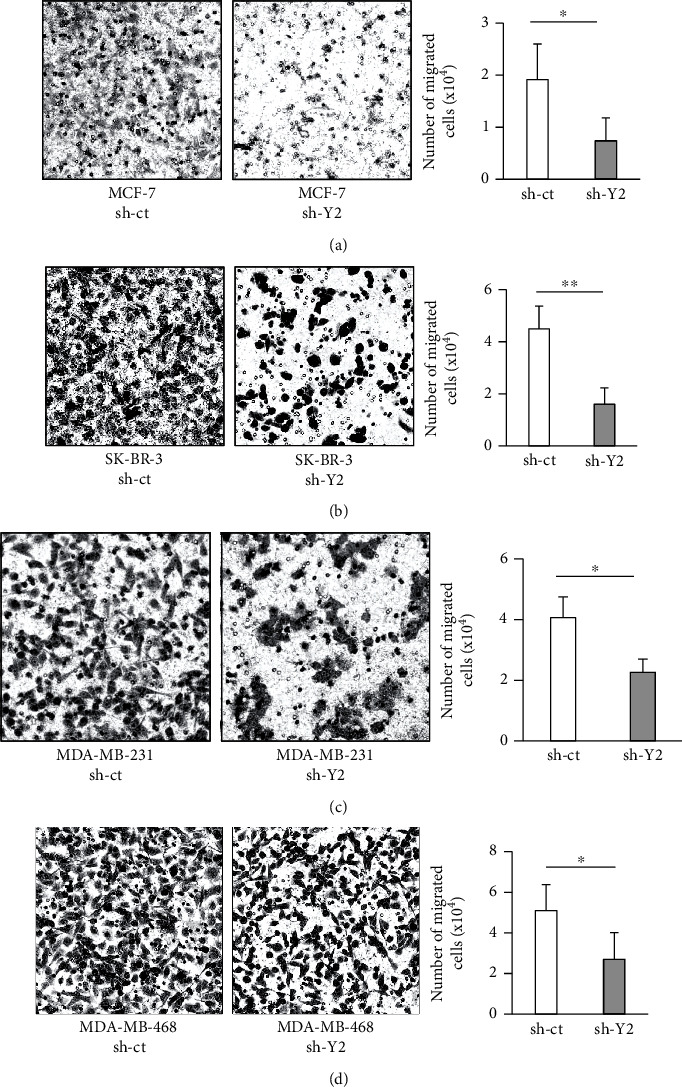
Effect of YTHDC2 knockdown on breast cancer cell metastasis. Representative images of migrated cells under the membrane of transwell: (a) MCF-7; (b) SK-BR-3; (c) MDA-MB-231; (d) MDA-MB-468. The data of bar graph are represented as the mean ± SD of the number of migrated cells, and the results were analyzed using Student's *t*-test. ^∗^*P* < 0.05 and ^∗∗^*P* < 0.01 versus sh-ct.

**Figure 5:**
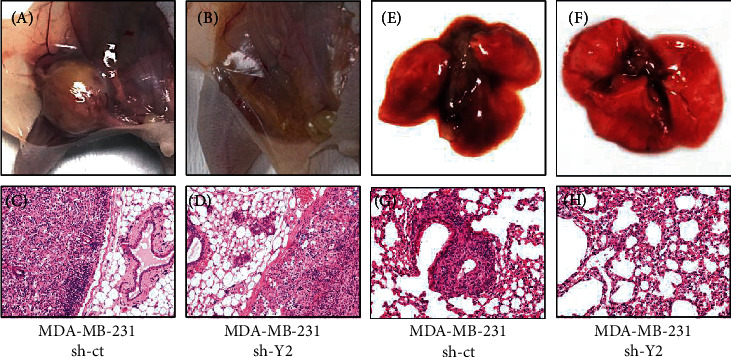
Effect of YTHDC2 knockdown on cancer growth and metastasis in vivo. Representative gross pathology and histopathology of mammary gland derived from MDA-MB-231 (a, c) sh-ct and (b, d) sh-Y2 injected mouse. Representative gross pathology and histopathology of lung derived from MDA-MB-231 (e, g) sh-ct and (f, h) sh-Y2 injected mouse.

To examine the effect of YTHDC2 knockdown on the metastatic ability of the cells, we performed a transwell assay. Similar with the sphere-forming ability, the metastatic ability was significantly reduced in all of the YTHDC2-knocked-down cells (Figures 4(a)–4(d)). We further investigated the metastatic ability of YTHDC2-knocked-down breast cancer cells *in vivo*. We transplanted control and YTHDC2-knocked-down MDA-MB-231 cells into mouse mammary glands and evaluated the lung metastatic potential of the transplanted cells. At 6 weeks after transplantation, the nude mice were sacrificed, and lung metastasis was examined by histopathology. The sizes of the breast tumors in the mammary glands were clearly larger in the control cell-transplanted group than in the YTHDC2-knocked-down cell-transplanted group (Figures 5(a) and 5(b)). Furthermore, lung metastasis was observed in five of the six mice injected with the control cells, whereas only one of the six mice injected with the YTHDC2-knocked-down cells showed lung metastasis (Figures 5(c) and 5(d) and Table 2).

### 3.4. The Effect of YTHDC2 Knockdown on the Expression of Stemness and EMT Marker Is Different between the Cell Lines

**Figure 6:**
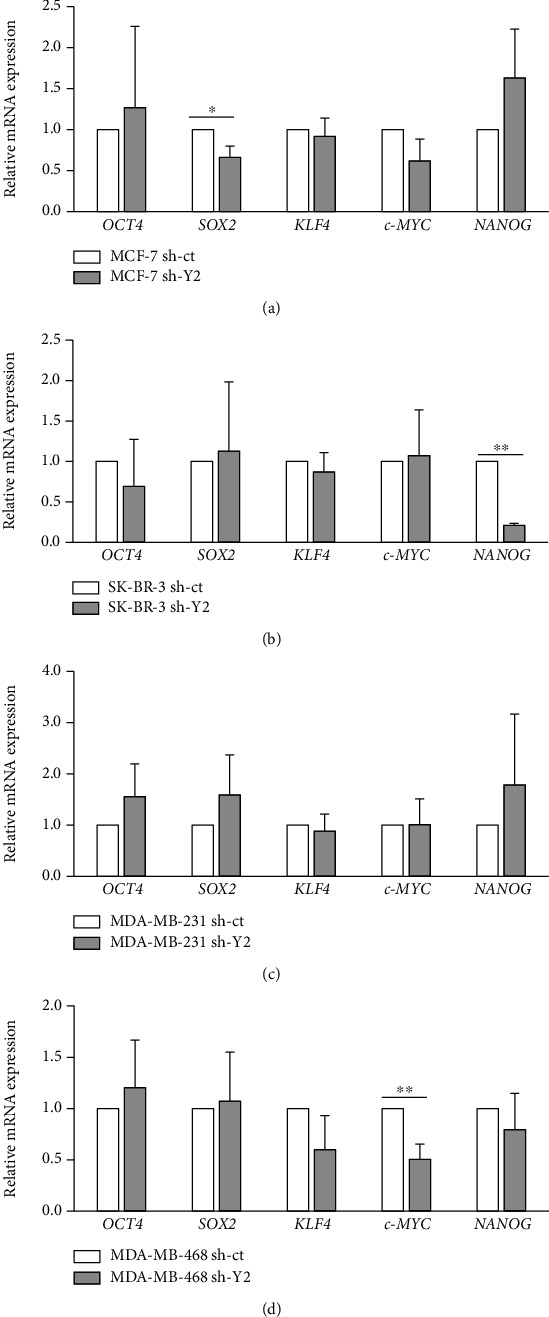
Effect of YTHDC2 knockdown on the expression of stem cell markers. Relative mRNA expression levels of OCT4, SOX2, KLF4, c-MYC, and NANOG: (a) MCF-7; (b) SK-BR-3; (c) MDA-MB-231; (d) MDA-MB-468. The data of the bar graph are represented as the mean ± SD of the number of migrated cells, and the results were analyzed using Student's *t*-test. ^∗^*P* < 0.05 and ^∗∗^*P* < 0.01 versus sh-ct.

**Figure 7:**
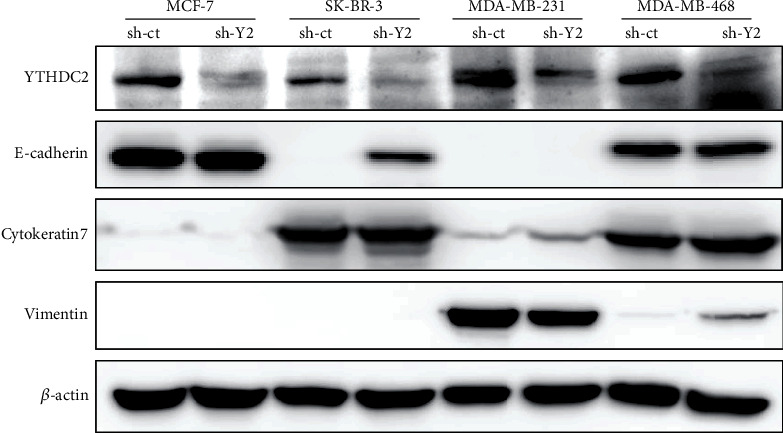
Effect of YTHDC2 knockdown on the expression of EMT markers. Representative protein levels of YTHDC2, E-cadherin, cytokeratin 7, and vimentin: (a) MCF-7; (b) SK-BR-3; (c) MDA-MB-231; (d) MDA-MB-468.

The sphere-forming and metastatic ability of breast cancer cells was significantly suppressed by the YTHDC2 knockdown, suggesting that the expression of genes related to stemness and metastasis was downregulated in the YTHDC2-knocked-down cells. Therefore, we firstly examined the mRNA expression of stemness markers, including *OCT4*, *SOX2*, *c-MYC*, *KLF4*, and *NANOG* gene, in breast cancer cells by qRT-PCR analysis (Figures 6(a)–6(d)). Although the expression of some stemness markers was reduced in several cell lines, the reduced genes were differed for each cell line. Next, we examined the expression of several EMT markers by western blot analysis (Figure 7). We then observed that the expression levels of mesenchymal marker vimentin were downregulated in the YTHDC2-knocked-down MDA-MB-231 cells, while those of the epithelial marker cytokeratin 7 were upregulated (Figure 7). However, the expression of vimentin was upregulated in the YTHDC2-knocked-down MDA-MB-468 cells (Figure 7). In addition, E-cadherin expression was upregulated only in YTHDC2-knocked-down SK-BR-3 cells (Figure 7). These results suggested that the roles of YTHDC2 are different for each breast cancer cell line. However, all of these genes that were altered by YTHDC2 knockdown are involved in cancer stemness and metastasis, suggesting that YTHDC2 plays important roles in malignant progression of breast cancer cells.

## 4. Discussion

In the present study, the YTHDC2 expression level showed a positive correlation with the stage of the breast cancers, suggesting that YTHDC2 is involved in the progression of breast cancer. The regulatory mechanism of *YTHDC2* expression has been investigated in several studies. Zhou et al. recently reported that *YTHDC2* expression is induced by starvation stress and is decreased by a high-fat diet in mouse liver [12]. Epigenetic modifications of chromatin and genomic DNA are suggested to be involved in *YTHDC2* gene expression. We previously reported that the inhibition of histone deacetylase activity suppressed the transcription of *YTHDC2* in HCC [23]. DNA methylation is also thought to be involved in the regulation of *YTHDC2* gene expression. He et al. recently reported that DNA methylation of the promoter region suppressed *YTHDC2* expression in nasopharyngeal carcinoma [14]. In contrast, Yang et al. reported that the frequency of DNA methylation in the 3′ untranslated region was positively correlated with the *YTHDC2* expression level in breast cancers [24]. To clarify the role of YTHDC2 in cancer, it is necessary to elucidate the epigenetic modification mechanisms involved in the regulation of *YTHDC2* gene expression.

In the present study, we knocked down YTHDC2 expression in breast cancer cell lines including MCF-7, SK-BR-3, MDA-MB-231, and MDA-MB-468 and found that knockdown of YTHDC2 suppressed the sphere-forming and metastatic ability of them. These results are consistent with our previous studies in CRC and HCC [13, 23]. Although the mRNA expression of stemness markers, such as SOX2, c-MYC, and NANOG, was reduced by YTHDC2 knockdown, the alteration of gene expression pattern was not the same for all of breast cancer cell lines. Similar with stemness markers, the expression of EMT markers and the cell proliferation rate in normal culture condition also showed different changes between the cell lines. These results suggest that YTHDC2 has different roles for each breast cancer cell line.

Several studies have demonstrated that YTHDC2 binds to m^6^A-modifed mRNAs by YTH domain [4, 5, 7, 8]. However, the binding targets of YTHDC2 differed between studies. In addition, even in the case of the same tissues, the binding targets of YTHDC2 are altered by their situation. Recently, an interesting study about the targets of YTHDC2 has been reported by Saito et al. [25]. In their study, they showed that the binding targets of YTHDC2 in mouse testis are altered dramatically during the development of the testis. These studies suggest that YTHDC2 target mRNAs may also change when stages and subtypes of breast cancer are different. The function of YTHDC2 is generally thought to be the degradation of target mRNAs and the regulation of translation efficiency of them. For example, YTHDC2 has been shown to be enhance the degradation of several mRNAs that are involved in meiosis by binding to the m^6^A-modified 3′ untranslated region [5, 7]. In other studies, YTHDC2 has been shown to regulate the translation of some genes, such as IGF1R and HCV, by eliminating the secondary structure of its mRNA [4, 11, 14]. On the other hand, some studies reported that YTHDC2 is also involved in the stability of m^6^A-modified mRNAs, such as meiosis-related genes and *interleukin-6* gene [26, 27]. m^6^A modification has been found in *SOX2*, *c-MYC*, and *NANOG* mRNAs and is thought to regulate the stabilization/destabilization and translation of them [28–30]. YTHDC2 may be involved in the regulation process of these genes via m^6^A modification. Further studies are required to elucidate in detail the mechanism by which YTHDC2 regulates these transcription factors.

In summary, our findings demonstrated that YTHDC2 contributes to breast cancer progression by regulating the expression of stemness transcription factors, indicating the potential of YTHDC2 as a therapeutic target for breast cancer patients.

## Figures and Tables

**Table 1:** Association between YTHDC2 expression and clinicopathological parameters.

Variable	*n*	YTHDC2 expression				*P* value
		0	1+	2+	3+	
All cases	99	28	36	15	20	
Age (years)						
≤60	43	14	14	9	6	0.413^†^
>60	56	14	22	6	14	
Stage						
I	49	17	18	9	5	0.032^‡^
IIA	30	7	11	3	9	
IIB	6	3	1	0	2	
IIIA	1	0	1	0	0	
IIIB	6	1	3	1	1	
IIIC	3	0	1	2	0	
IV	4	0	1	0	3	
Subtype						
Luminal A	31	11	8	6	6	0.882^†^
Luminal B	46	10	20	8	8	
HER2 enriched	9	2	4	0	3	
Triple negative	13	5	4	1	3	
ER						
Negative	22	7	8	1	6	0.878^†^
Positive	77	21	28	14	14	
PgR						
Negative	38	12	16	3	7	0.295^†^
Positive	61	16	20	12	13	
HER2						
Negative	85	23	30	15	17	0.402^†^
Positive	14	5	6	0	3	

^†^Determined using the Kruskal–Wallis test; ^‡^determined using the Spearman rank correlation coefficient.

**Table 2:** Frequency of lung metastasis following intramammary gland injection to nude mice.

	MDA-MB-231 sh-ct	MDA-MB-231 sh-Y2
Primary tumor	6/6	4/6
Lung metastasis	5/6	1/6

## Data Availability

The data presented in this study are available on request from the corresponding author.

## Supplementary Materials

Supplementary Materials Supplementary Figure 1: effect of YTHDC2 knockdown on cell proliferation of breast cancer cells. The data of line graphs are represented as the mean ± SD of the number of cells: (A) MCF-7; (B) SK-BR-3; (C) MDA-MB-231; (D) MDA-MB-468. The results were analyzed using two-way analysis of variance followed by the Tukey-Kramer post hoc test. ^∗∗^*P* < 0.01 versus sh-ct.
