# Supplementary material for: YTHDC2 Promotes Malignant Phenotypes of Breast Cancer Cells
Source: J Oncol. 2022 Oct 7;2022:9188920. doi: 10.1155/2022/9188920 (PMC9568362; doi:10.1155/2022/9188920)
Supplement: Supplementary Materials — Supplementary Figure 1: effect of YTHDC2 knockdown on cell proliferation of breast cancer cells. The data of line graphs are represented as the mean ± SD of the number of cells: (A) MCF-7; (B) SK-BR-3; (C) MDA-MB-231; (D) MDA-MB-468. The results were analyzed using two-way analysis of variance followed by the Tukey-Kramer post hoc test. ∗∗P < 0.01 versus sh-ct. [file 9188920.f1.pptx]

## Slide 1
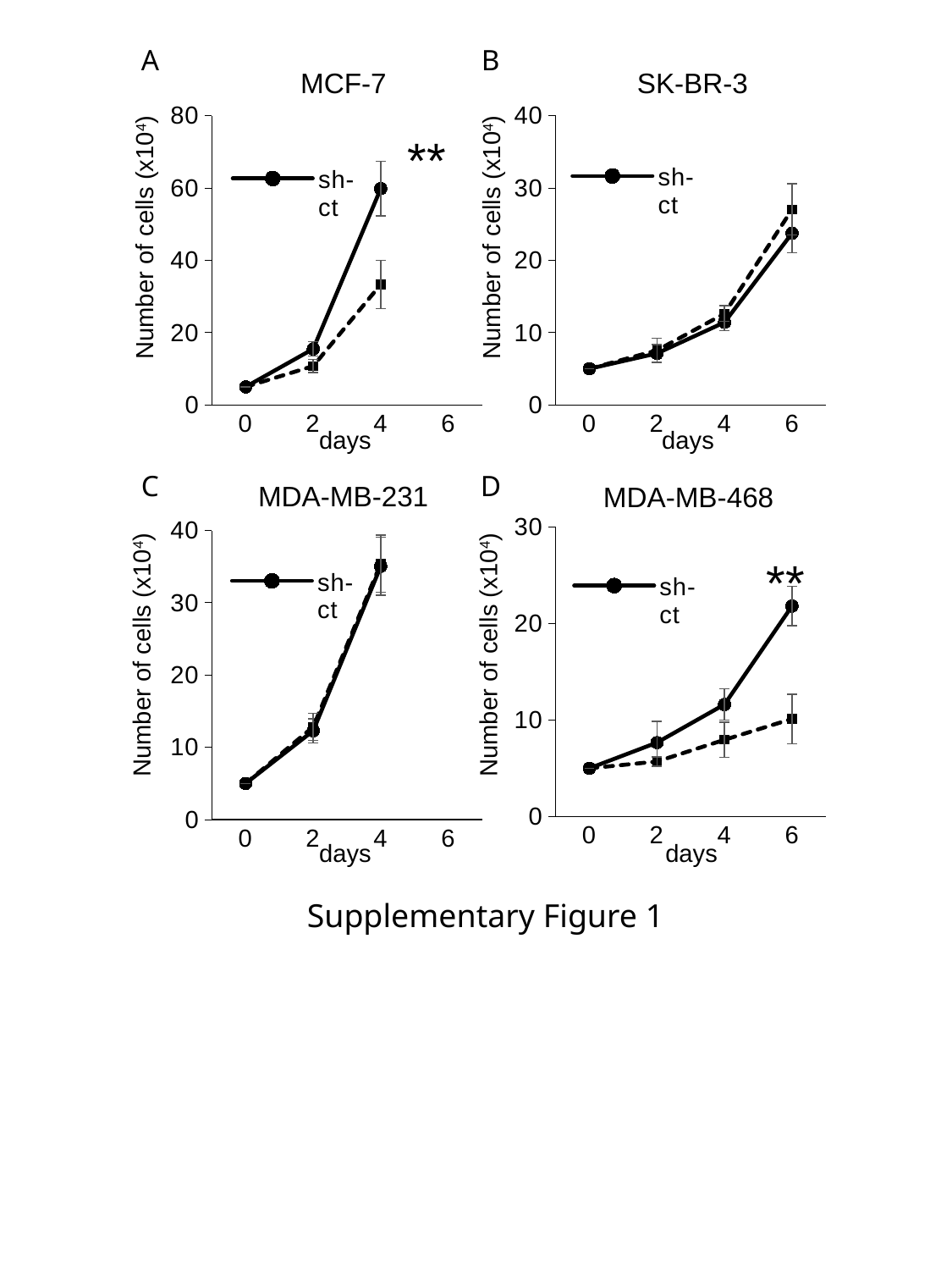

A
B
MCF-7
SK-BR-3
[unsupported chart]
### Chart
| Category | | sh-Y2 |
|---|---|---|
| 0 | 5.0 | 5.0 |
| 2 | 7.125 | 7.541666666666667 |
| 4 | 11.416666666666666 | 12.625 |
| 6 | 23.75 | 27.041666666666668 |**
Number of cells (x104)
Number of cells (x104)
days
days
C
D
MDA-MB-231
MDA-MB-468
### Chart
| Category | | sh-Y2 |
|---|---|---|
| 0 | 5.0 | 5.0 |
| 2 | 7.666666666666667 | 5.708333333333333 |
| 4 | 11.625 | 7.958333333333333 |
| 6 | 21.833333333333332 | 10.125 |
[unsupported chart]
**
Number of cells (x104)
Number of cells (x104)
days
days
Supplementary Figure 1
